# ﻿Morphological characteristics and phylogenetic analyses revealed four new species (Basidiomycota) in the Yunnan-Guizhou Plateau, China

**DOI:** 10.3897/mycokeys.113.140932

**Published:** 2025-02-12

**Authors:** Siyuan He, Lu Wang, Kaize Shen, Hongmin Zhou

**Affiliations:** 1 College of Forestry, Southwest Forestry University, Kunming 650224, China; 2 Yunnan Key Laboratory of Gastrodia and Fungi Symbiotic Biology, Zhaotong University, Zhaotong 657000, China; 3 The Key Laboratory of Forest Resources Conservation and Utilization in the South-west Mountains of China Ministry of Education, Key Laboratory of National Forestry and Grassland Administration on Biodiversity Conservation in Southwest China, Yunnan Provincial Key Laboratory for Conservation and Utilization of In-forest Resource, Southwest Forestry University, Kunming 650224, China

**Keywords:** 4 new taxa, Agaricales, biodiversity, Russulales, taxonomy, Yunnan province

## Abstract

Four new fungi, *viz. Clavulinopsiswumengshanensis* (Clavariaceae, Agaricales), *Henningsomycesbambusae* (Porotheleaceae, Agaricales), *Xenasmabisterigmatae*, and *X.guttulata* (Xenasmataceae, Russulales), from Yunnan Province in China, are proposed, based on a combination of morphological features and molecular evidence. Phylogenetic analyses were conducted using a combined dataset of internal transcribed space and nuclear ribosomal RNA large subunit sequences. The ITS+LSU analysis showed that *Clavulinopsiswumengshanensis***sp. nov.** groups with *C.aurantiocinnabarina*, *Henningsomycesbambusae***sp. nov.** forms a sister group with *H.candidus*, *Xenasmabisterigmatae***sp. nov.** is clustered with *X.rimicola*, and *X.guttulata***sp. nov.** is clustered with *X.pruinosum.* The morphology and multi-gene phylogenetic analyses confirmed the placement of the four new taxa. *Clavulinopsiswumengshanensis* is distinguished by buff-yellow to straw-yellow basidiomata, clavate to subcylindrical basidia with four sterigmata, and subglobose basidiospores with several guttules (7–8.5 × 6–7.5 µm); *Henningsomycesbambusae* is characterized by white to cream basidiomata with short cylindric to tubular colonies, cylindrical to subcylindrical basidia with two sterigmata and globose to subglobose basidiospores (6.5–8.5 × 6.5–8.5 µm); *Xenasmabisterigmatae* can be characterized by its membranaceous and ash-grey basidiomata, clavate to subcylindrical basidia with two sterigmata and ellipsoid to subglobose basidiospores (10–12.5 × 8–10.5 µm); *X.guttulata* is distinguished by membranaceous and white to cream basidiomata, clavate to subcylindrical basidia with two sterigmata and ellipsoid to narrowly ellipsoid basidiospores (7–9 × 5.5–7.5 µm).

## ﻿Introduction

The phylum Basidiomycota R.T. Moore represents one of the major divisions in the fungal tree of life, with global estimates that it encompasses 1.4–4.2 million species in the phylum and the latest estimates of 0.7 to 1 million species, which represent about 28–40% of all fungal diversity (Hyde 2022; He et al. 2024; Liu et al. 2024). The basidiomata of Basidiomycota exhibit complex forms, such as coralloid, corticioid, gilled, hydnoid, poroid, and toothed basidiomata (Bernicchia and Gorjón 2010; He et al. 2019; Zhou et al. 2024a). Traditionally, Basidiomycota is typically characterized by the basidia and basidiospores and some variable morphological characters, such as diverse cellular constructions in hyphal systems and meiosporangia; the basidiomata reflect a profound evolutionary history at the various taxonomic levels within Basidiomycota (Zhao et al. 2016; Hyde et al. 2023; Dong et al. 2024). Nowadays, DNA sequence-based classification and identification have become the standard methodology in fungal taxonomy (Dai et al. 2021; Zhang et al. 2023; Dong et al. 2024; He et al. 2024).

The genus *Clavulinopsis* Overeem, classified within the family Clavariaceae (Agaricales, Basidiomycota), includes species that are widely distributed. It is characterized by yellow, orange, or creamy white basidiomata, with simple or regularly dichotomously branched, cylindric or fusoid stems; generative hyphae with obtuse, occasionally inflated hyphae with clamp connections; 2–4-spored basidia; and smooth or echinulate basidiospores occasionally with big guttules (Petersen 1996; Knudsen and Vesterholt 2012; Keleş and Kaya 2021; Yan et al. 2023). More than 171 taxon records of the genus currently are listed in the Index Fungorum database (http://www.indexfungorum.org; accessed on 2 November 2024), and approximately 84 species names are legitimately published (Yan et al. 2023). However, the phylogeny of *Clavulinopsis* is ambiguous due to a lack of molecular evidence and morphological data. Two similar genera, *Clavaria* and *Ramariopsis*, are easily confused with *Clavulinopsis* in clavarioid basidiomata. However, the micromorphology feathers are different between the three genera; *viz.*, *Ramariopsis* has various basidiomata, but those in *Clavaria* and *Clavulinopsis* are simple; clamp connections are present on the basidia and hyphae in *Clavulinopsis* (Corner 1950, 1970; Petersen and Corner 1968; Yan et al. 2023). Furthermore, the outline of *Clavulinopsis* was defined by the basidiospore ornamentation, and the related taxa were examined by D.N. Pegler and T.W.K. Young. Applying the molecular phylogenetic methods generated considerable promotion in the generic definition of *Clavulinopsis*, gradually clarifying the classification boundaries among several closely related genera, particularly *Clavaria* and *Ramariopsis* (Furtado et al. 2016; Yan et al. 2023; He et al. 2024).

*Henningsomyces* Kuntze, a type of cyphelloid fungi first described by Kuntze (1898), belongs to the family Porotheleaceae (Agaricales) (He et al. 2024). Previously, cyphelloid fungi were classified in the family Cyphellaceae based on similar morphology. Subsequently, the name Porotheleaceae (Murrill 1916) was later established for this group (Cooke 1957, 1961). However, the phylogenetic relationship of many cyphelloid fungi remains ambiguous. The genus *Henningsomyces*, a typical cyphelloid fungus with cylindric basidiomata, is typified by *H.candidus* (Pers.) Kuntze and is characterized by annual basidiomata consisting of sparse or gregarious tubes, a monomitic hyphae system that typically exhibits both clamp connections and simple septa, an absence of cystidia, and globose to subglobose basidiospores (Wei and Qin 2009; Liu et al. 2023). Among the 50 records of the genus currently listed in the Index Fungorum database (http://www.indexfungorum.org; accessed on 2 November 2024), approximately 22 species names are legitimately published (Liu et al. 2023). Among those studies, *Henningsomyces* forms a monophyletic lineage and nests into the order Agaricales (Moncalvo et al. 2006, Bodensteiner et al. 2004, Binder et al. 2005, Thorn et al. 2005, Baltazar et al. 2015, Lucas and Dentinger 2015, Moreno et al. 2017). However, the placement of *Henningsomyces* and related taxa has not yet been substantiated. In recent years, based on morphological examination and molecular phylogenetic analysis, six new species were described in China (Yan et al. 2023). Most mycologists focused on the poroid or corticioid species, but the cyphelloid species bearing cup-, bowl-, or tube-shaped “cyphelloid” hymenophores were rarely reported, such as the genus *Henningsomyces* bearing the cylindric basidiomata (Wei and Qin 2009).

*Xenasma* Donk, classified in the family Xenasmataceae (Russulales, Basidiomycota), was introduced in 1957 and is typified by *X.rimicola* (P. Karst.) Donk (Liberta 1960; Bernicchia and Gorjón 2010). This genus is characterized by the resupinate and smooth basidiomata, a monomitic hyphal system with clamps, generative hyphae, and globose to cylindrical, striate basidiospores (Liberta 1960; Bernicchia and Gorjón 2010). Among the 39 records of the genus currently listed in the Index Fungorum database (http://www.indexfungorum.org; accessed on 2 November 2024), only 11 species names are legitimately published (Bernicchia and Gorjón 2010; He et al. 2024). *Xenasma* Donk continues to intrigue mycologists due to its unique morphological characteristics and ecological roles (Bernicchia and Gorjón 2010). The genus is primarily found in temperate forest ecosystems, often growing on decaying wood and contributing to wood decomposition (Liu et al. 2024). Recent phylogenetic studies have expanded the understanding of its evolutionary relationships within the Russulales, highlighting the potential for undiscovered species in understudied regions (He et al. 2019, 2024). The morphology of the basidiome and hymenophore, together with habitat, are often regarded as important characters for the taxonomy of the order Russulales, and Xenasmataceae is the only family in which the smooth hymenophore configuration could be found (Larsson and Larsson 2003; Miller et al. 2006; He et al. 2024). In the latest study, there are only two genera in the family Xenasmataceae, namely *Xenasma* and *Xenosperma* Oberw, in which the genus *Xenasma* is a mystery genus, and no new taxon was reported from this genus for nearly half a century (He et al. 2024).

The specimens of the three genera collected in the Yunnan-Guizhou Plateau, China, which could not be assigned to any described species of the order. Therefore, four new species, *viz. Clavulinopsiswumengshanensis*, *Henningsomycesbambusae*, *Xenasmabisterigmatae*, and *X.guttulate*, are proposed with descriptions, illustrations, and phylogenetic analysis results.

## ﻿Materials and methods

### ﻿Morphology

Fresh fruiting bodies of the fungi were collected from Wumengshan National Nature Reserve in Zhaotong of Yunnan Province, China, and the important collection information was recorded (Rathnayaka et al. 2024). Specimens were dried in an electric food dehydrator at 40 °C (Hu et al. 2022), then sealed and stored in an envelope bag and deposited in the herbarium of the Southwest Forestry University (SWFC), Kunming, Yunnan Province, China. Macromorphological descriptions are based on field notes and photos captured in the field and lab. Color terminology follows Petersen (1996). Micromorphological data were obtained from the dried specimens when observed under a light microscope following the previous study (Zhao et al. 2023; Zhou et al. 2024b). The following abbreviations are used: KOH = 5% potassium hydroxide water solution, CB = Cotton Blue, CB– = acyanophilous, IKI = Melzer’s Reagent, IKI– = both inamyloid and indextrinoid, Lm = mean spore length (arithmetic average for all spores), Wm = mean spore width (arithmetic average for all spores), Q = variation in the L/W ratios between the specimens studied, and n = a/b (number of spores (a) measured from given number (b) of specimens).

### ﻿Molecular phylogeny

The CTAB rapid plant genome extraction kit-DN14 (Aidlab Biotechnologies Co., Ltd., Beijing) was used to obtain DNA from dried specimens, and PCR was performed according to the manufacturer’s instructions with some modifications. ITS locus was amplified using the primer pairs ITS5/ITS4 (White et al. 1990). The nuclear LSU region was amplified with primer pair LR0R and LR7 (Vilgalys and Hester 1990). The PCR procedure for ITS was as follows: initial denaturation at 95 °C for 3 min, followed by 35 cycles at 94 °C for 40 s, 54 °C for 45 s, and 72 °C for 1 min, and a final extension at 72 °C for 10 min. The PCR procedure for LSU was as follows: initial denaturation at 94 °C for 1 min, followed by 35 cycles at 94 °C for 30 s, 50 °C for 1 min, and 72 °C for 1.5 min, and a final extension at 72 °C for 10 min. All newly generated sequences were submitted to GenBank and are listed in Table 1.

**Table 1.** Names, voucher numbers, references, and corresponding GenBank accession numbers of the taxa used in the phylogenetic analyses. [* Indicates type materials;—indicates sequence unavailability].

Taxa	Locality	Voucher no.	GenBank accession no.		References
			ITS	28S	
*Clavariaapulica*	Italy	AMB 150	MT853065	MT853066	Agnell and Papetti 2020
*Clavulinopsisamoena*	Australia	PBM3381	—	HQ877702	Hyde et al. 2016
*Clavulinopsisaspersa*	China	MHHNU10153	OQ703777	OQ703794	Yan et al. 2023
*Clavulinopsisaspersa*	China	MHHNU10342*	OQ703778	OQ703795	Yan et al. 2023
*Clavulinopsisaurantiaca*	Brazil	URM<BRA>:84212*	—	KX227749	Hyde et al. 2016
*Clavulinopsisaurantiaca*	Brazil	URM<BRA>:84216	KC348464	NG058946	Hyde et al. 2016
*Clavulinopsisbicolor*	China	MHHNU10381*	OQ703780	OQ703797	Yan et al. 2023
*Clavulinopsisbispora*	China	MHHNU11188	OQ703782	OQ703799	Yan et al. 2023
*Clavulinopsisbispora*	China	MHHNU11181*	OQ703781	OQ703798	Yan et al. 2023
*Clavulinopsiscorallinorosacea*	Australia	PBM3380	KP257144	HQ877707	Hyde et al. 2016
*Clavulinopsiscorniculata*	USA	TENN064106	KP257145	HQ877713	Hyde et al. 2016
*Clavulinopsiserubescens*	China	MHHNU10290	OQ703784	OQ703801	Yan et al. 2023
*Clavulinopsiserubescens*	China	MHHNU8040*	OQ703783	OQ703800	Yan et al. 2023
*Clavulinopsisfusiformis*	USA	PBM 2804	—	EF535273	Hyde et al. 2016
*Clavulinopsisfusiformis*	USA	TENN064110	—	HQ877717	Hyde et al. 2016
*Clavulinopsisgracillima*	Canada	MO 215748	KY706170	—	Hay et al. 2019
*Clavulinopsisincarnata*	China	MHHNU11331	OQ703788	OQ703805	Yan et al. 2023
*Clavulinopsisincarnata*	China	MHHNU11330*	OQ703787	OQ703804	Yan et al. 2023
*Clavulinopsismiyabeana*	China	ZP-2118	MK427059	—	Chen and Zhang 2019
*Clavulinopsissulcata*	Australia	PBM3379	—	HQ877709	Hyde et al. 2016
*Clavulinopsissulcata*	New Zealand	PDD78241	—	DQ284904	Dentinger and McLaughlin 2006
*Clavulinopsistrigonospora*	China	MHHNU9186	OQ703789	OQ703806	Yan et al. 2023
*Clavulinopsistrigonospora*	Italy	AMB: 18557*	NR176720	NG088120	Franchi and Marchetti 2021
*Clavulinopsistropicalis*	China	MHHNU10721	OQ703792	OQ703809	Yan et al. 2023
*Clavulinopsistropicalis*	China	MHHNU10722*	OQ703793	OQ703810	Yan et al. 2023
***Clavulinopsiswumengshanensis***	**China**	**CLZhao 29651**	**PQ408630**	**PQ408635**	**Present Study**
***Clavulinopsiswumengshanensis***	**China**	CLZhao 29612*	**PQ408629**	**PQ408634**	**Present Study**
*Clitocybulafamilia*	Slovakia	BRNM 736053	JF730328	JF730323	Antonín et al. 2011
*Clitocybulaintervenosa*	São Tomé	BAP 613 SFSU*	MH414561	MH385335	Cooper 2018
*Clitocybulalacerata*	Italy	AMB 18779	OM422757	OM423633	Consiglio et al. 2022
*Clitocybulalacerata*	Czech Republic	PRM 915404	LT854054	LT854030	Antonín et al. 2019
*Clitocybulaoculus*	USA	PBM 1156	DQ192178	DQ151452	Matheny et al. 2006
*Gerronemakeralense*	India	CAL 1666*	NR159832	NG064531	Latha et al. 2018
*Gerronemakuruvense*	India	CAL 1665*	NR159831	NG064530	Latha et al. 2018
*Gerronemaxanthophyllum*	Czech Republic	PRM 924657	LT854023	LT854023	Antonín et al. 2019
***Henningsomycesbambusae***	**China**	**CLZhao 33024**	**PQ408626**	—	**Present study**
***Henningsomycesbambusae***	**China**	**CLZhao 33085**	**PQ408627**	—	**Present study**
***Henningsomycesbambusae***	**China**	CLZhao 33088*	**PQ408628**	—	**Present study**
*Henningsomycescandidus*	France	PB338	AY571044	AY571008	Bodensteiner et al. 2004
*Henningsomycescandidus*	Canada	T156	AY571043	—	Bodensteiner et al. 2004
*Henningsomyceshengduanensis*	China	LWZ 20190807-22b	OR557251	OR527277	Liu et al. 2024
*Henningsomyceshengduanensis*	China	LWZ 20190807-11b*	OR557250	OR527276	Liu et al. 2024
*Hydropodiasubalpina*	Italy	AMB 18784	OM422761	OM423638	Consiglio et al. 2022
*Hydropodiasubalpina*	Italy	AMB 18785	OM422762	OM423639	Consiglio et al. 2022
*Hydropodiasubalpina*	Turkey	OKA TR-K364	MN701620	MN700170	Kaygusuz et al. 2020
*Leucoinocybelenta*	Italy	AMB 18837	OM422765	OM423643	Consiglio et al. 2022
*Leucoinocybetaniae*	Italy	AMB 18838	OM422766	OM423644	Consiglio et al. 2022
*Leucoinocybetaniae*	Italy	AMB 18839	OM422767	OM423645	Consiglio et al. 2022
*Porotheleumalbodescendens*	New Zealand	PDD 96321*	OL998343	OL998382	Consiglio et al. 2022
*Porotheleumdomingense*	Dominican Republic	JBSD 131801*	OM422768	OM423646	Consiglio et al. 2022
*Porotheleumfimbriatum*	France	CBS 465.50	MH856711	—	Vu et al. 2019
*Porotheleumparvulum*	Dominican Republic	JBSD 131802*	OM422783	OM423657	Consiglio et al. 2022
*Pseudohydropuscommenticius*	New Zealand	PDD 86984*	OL998339	OL998379	Consiglio et al. 2022
*Pseudohydropusfloccipes*	Czech Republic	BRNM 816173	OM422758	OM423634	Consiglio et al. 2022
*Pseudohydropusparafunebris*	New Zealand	PDD 87227*	JQ694112	—	Consiglio et al. 2022
*Pterulaecho*	USA	AFTOL-ID 711	DQ494693	—	Matheny et al. 2006
*Radulomycescopelandii*	China	Dai 15061	KU535664	KU535672	Zhao et al. 2016
*Radulotubusresupinatus*	China	Cui 8383*	KU535660	KU535668	Zhao et al. 2016
*Rectipilusafibulatus*	UK	K(M)189533*	KT893457	—	Lucas and Dentinger 2015
***Xenasmabistaminatae***	**China**	CLZhao 32542*	**PQ408631**	—	**Present study**
***Xenasmabistaminatae***	**China**	**CLZhao 32600**	**PQ408632**	—	**Present study**
***Xenasmaguttulata***	**China**	CLZhao 32193*	**PQ408633**	—	**Present study**
*Xenasmapraeteritum*	USA	Alden Dirks:ACD0185	OM009268	—	Unpublished
*Xenasmapruinosum*	Japan	OTU1299	MT594801	—	Unpublished
*Xenasmarimicola*	Australia	N.L. Bougher NLB 1449	MT537020	—	Unpublished
*Xenasmarimicola*	Australia	N.L. Bougher NLB 1571	MT571671	—	Unpublished

Sequences generated for this study were aligned, with additional sequences downloaded from GenBank. Sequences were aligned using MAFFT v.7 (https://mafft.cbrc.jp/alignment/server/), adjusting the direction of nucleotide sequences according to the first sequence (accurate enough for most cases) and selecting the G-INS-i iterative refinement method (Katoh et al. 2019). Alignments were manually adjusted to maximize alignment and minimize gaps with BioEdit v.7.0.9 (Hall 1999). A dataset of concatenated ITS and LSU sequences was used to determine the phylogenetic position of the new species. Maximum likelihood (ML) analysis was performed using the CIPRES Science Gateway (Miller et al. 2010) based on the dataset using the RA × ML-HPC BlackBox tool, with the setting RA × ML halt bootstrapping automatically and 0.25 for maximum hours and obtaining the best tree using ML search. Other parameters in ML analysis followed default settings, and statistical support values were obtained using nonparametric bootstrapping with 1,000 replicates. Maximum parsimony (MP) analyses were applied to the combined three datasets following the methods outlined in a previous study (Zhao and Wu 2017), and the tree construction procedure was performed in PAUP* version 4.0b10 (Swofford 2002). Bayesian inference (BI) analysis based on the dataset was performed using MrBayes v.3.2.6 (Ronquist and Huelsenbeck 2003). The best substitution model for the dataset was selected by ModelFinder (Kalyaanamoorthy et al. 2017) using a Bayesian information criterion, and the model was used for Bayesian analysis. Four Markov chains were run from random starting trees. Trees were sampled every 1,000^th^ generation. The first 25% of sampled trees were discarded as burn-in, whereas other trees were used to construct a 50% majority consensus tree and for calculating Bayesian posterior probabilities (BPPs). The bootstrap support for ML is greater than or equal to 70%, and Bayesian posterior probabilities greater than or equal to 0.95 are indicated on the branches in the phylogenetic tree, respectively.

## ﻿Results

### ﻿The phylogeny of *Clavulinopsis*

**Figure 1.**
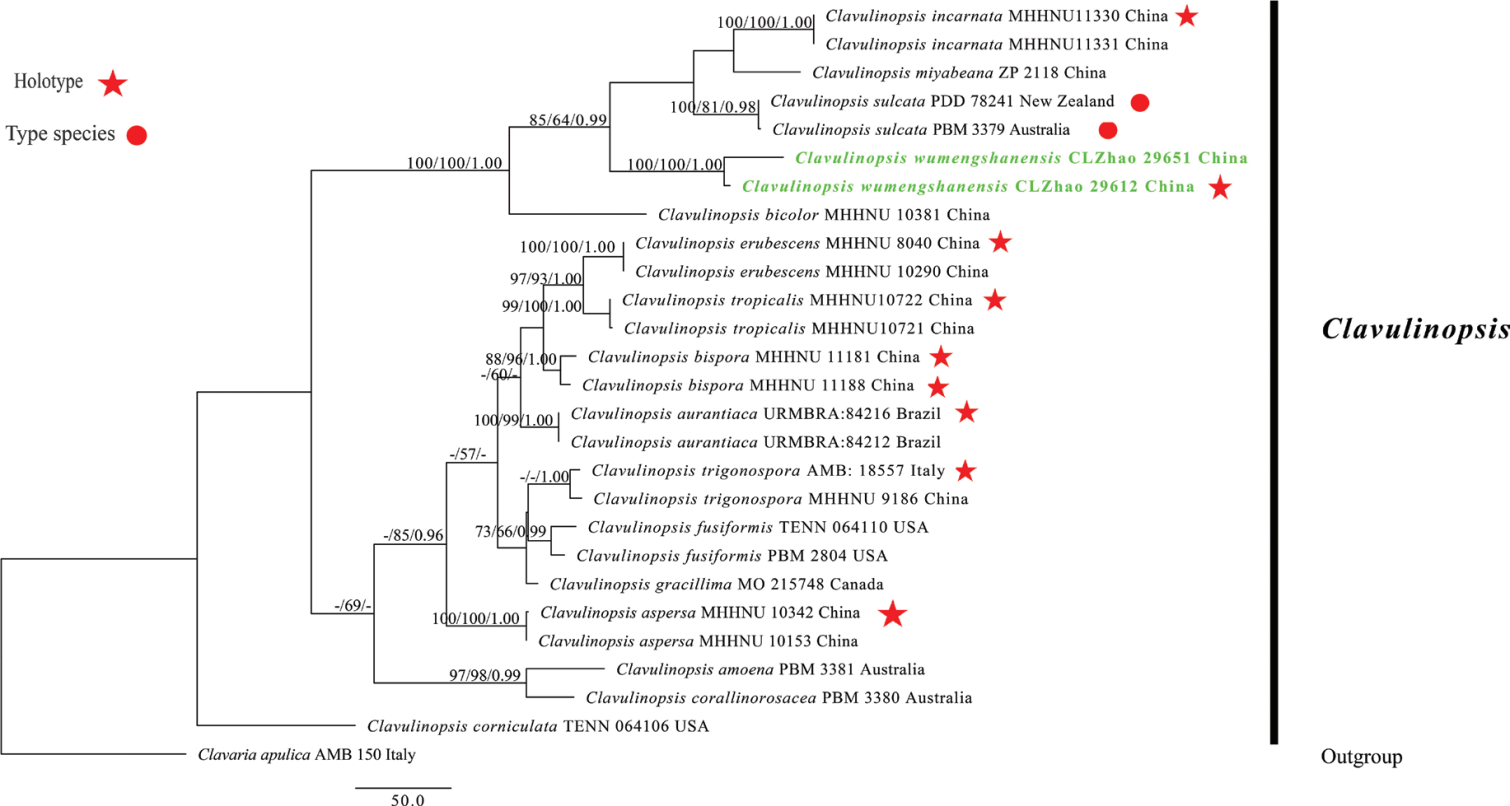
Phylogeny of species in *Clavulinopsis* generated by maximum likelihood based on ITS+LSU sequence data. Branches are labeled with maximum likelihood bootstrap ≥ 70%, a reduced lead value greater than 50%, and Bayesian posterior probabilities ≥ 0.95, respectively.

The dataset included ITS+LSU sequences from 27 samples representing 17 taxa. The datasets had an aligned length of 2,467 characters, of which 1,775 characters are constant, 273 are variable and parsimony-uninformative, and 419 are parsimony-informative. Maximum parsimony analysis yielded 1 equally parsimonious tree (TL = 1359, CI = 0.6799, HI = 0.5829, RI = 0.7129, RC = 0.4847). BI analysis yielded a similar topology to ML analysis, with an average standard deviation of split frequencies of 0.012252; trees were sampled every 1,000^th^ generation, 0.4 million in total. The effective sample size (ESS) for Bayesian analysis across the two runs is double the average ESS (avg ESS) = 201. Branches that received bootstrap support for ML ≥ 70%, MP ≥ 50%, and BI ≥ 0.95 were considered significantly supported, respectively. The ML tree was provided (Fig. 1). The phylogenetic tree (Fig. 1) reveals that the new species *Clavulinopsiswumengshanensis* is nested into the genus *Clavulinopsis* and has a close relationship with *C.aurantiocinnabarina* (Schwein.) Corner with full support (100/100/1.00).

### ﻿The phylogeny of *Henningsomyces*

**Figure 2.**
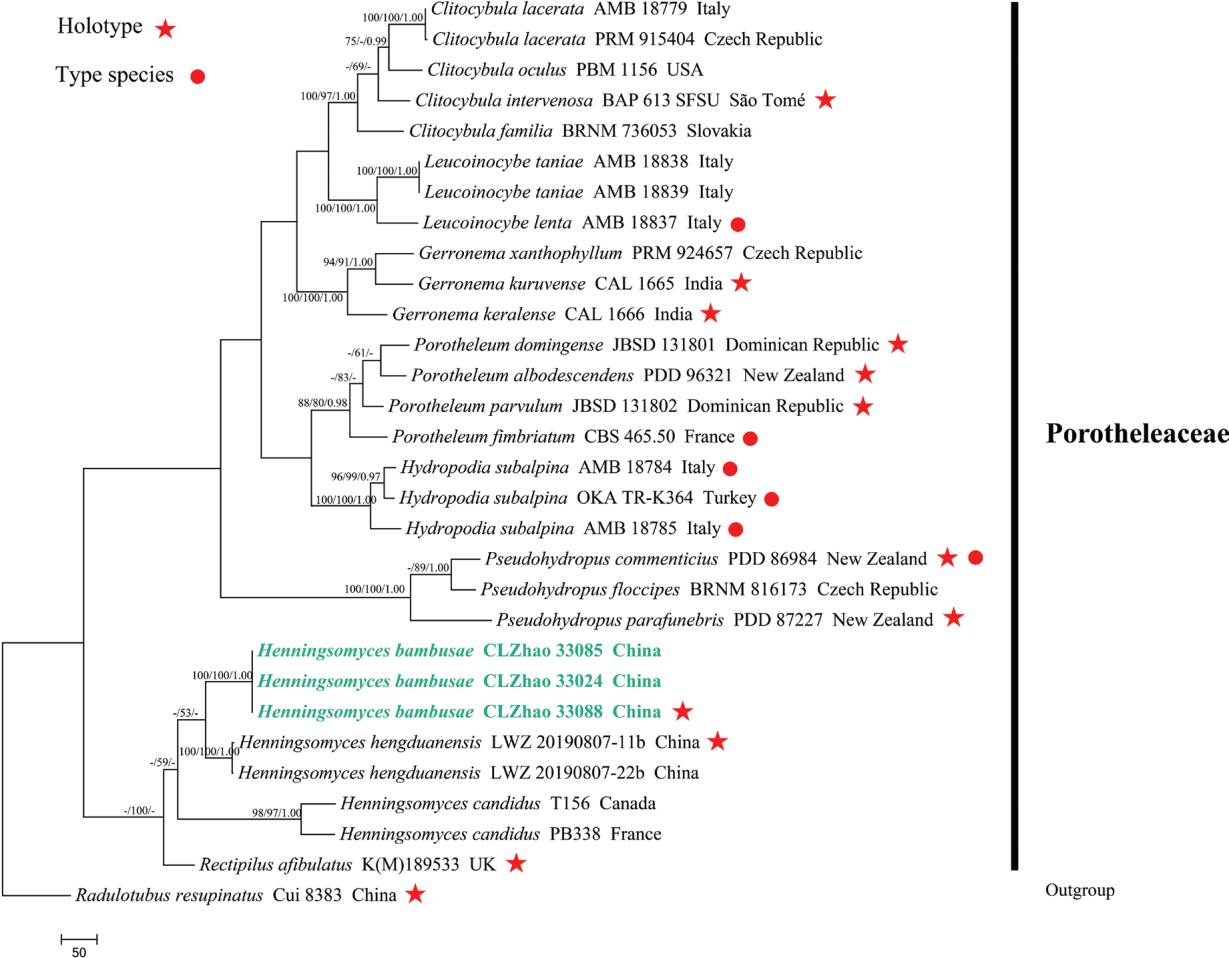
Phylogeny of species in *Henningsomyces* generated by maximum likelihood based on ITS sequence data. Branches are labeled with maximum likelihood bootstrap ≥ 70%, a reduced lead value greater than 50%, and Bayesian posterior probabilities ≥ 0.95, respectively.

The dataset included ITS sequences from 31 samples representing 23 taxa. The datasets had an aligned length of 2,375 characters, of which 1,204 characters are constant, 525 are variable and parsimony-uninformative, and 646 are parsimony-informative. Maximum parsimony analysis yielded 1 equally parsimonious tree (TL = 2,997, CI = 0.5706, HI = 0.4294, RI = 0.5875, RC = 0.3352). The BI analysis yielded a similar topology to the ML analysis, with an average standard deviation of split frequencies of 0.007176; trees were sampled every 1,000^th^ generation, 0.4 million in total. And the effective sample size (ESS) for Bayesian analysis across the two runs is double the average ESS (avg ESS) = 349. Branches that received bootstrap support for ML ≥ 70%, MP ≥ 50%, and BI ≥ 0.95 were considered significantly supported, respectively. The ML tree was provided (Fig. 2). The phylogenetic tree (Fig. 2) reveals the new species *Henningsomycesbambusae* nested into the genus *Henningsomyces* and has a close relationship with *H.candidus* (Pers.) Kuntze with full support (100/100/1.00).

### ﻿The phylogeny of *Xenasma*

**Figure 3.**
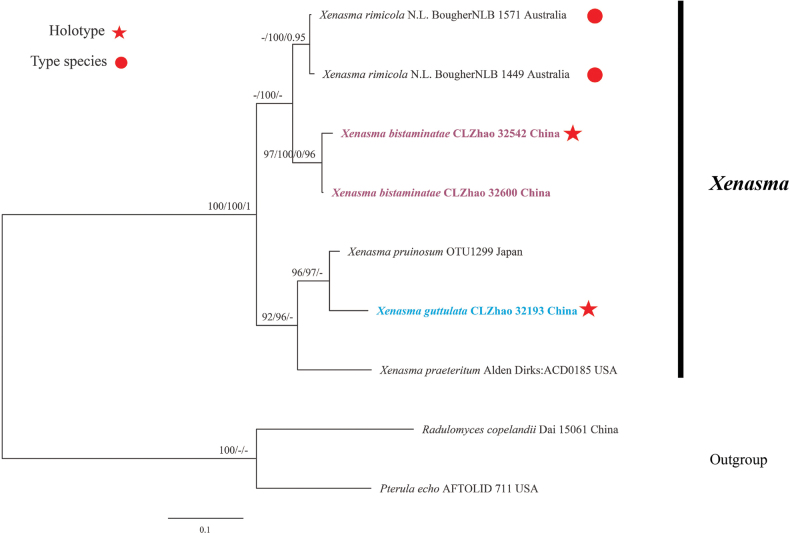
Phylogeny of species in *Xenasma* generated by maximum likelihood based on ITS sequence data. Branches are labeled with maximum likelihood bootstrap ≥ 70%, a reduced lead value greater than 50%, and Bayesian posterior probabilities ≥ 0.95, respectively.

The dataset included ITS sequences from nine samples representing seven taxa. The datasets had an aligned length of 944 characters, of which 574 characters are constant, 154 are variable and parsimony-uninformative, and 216 are parsimony-informative. Maximum parsimony analysis yielded 1 equally parsimonious tree (TL = 520, CI = 0.8942, HI = 0.1058, RI = 0.8243, RC = 0.7371). The BI analysis yielded a similar topology to the ML analysis, with an average standard deviation of split frequencies of 0.007297; trees were sampled every 1,000^th^ generation, 0.4 million in total. And the effective sample size (ESS) for Bayesian analysis across the two runs is double the average ESS (avg ESS) = 768.5. Branches that received bootstrap support for ML ≥ 70%, MP ≥ 50%, and BI ≥ 0.95 were considered significantly supported, respectively. The ML tree was provided (Fig. 3). The phylogenetic tree (Fig. 3) reveals the two new species, *Xenasmabisterigmatae* and *X.guttulate*, nested into the genus *Xenasma*. The taxon *X.bisterigmatae* has a close relationship with *X.rimicola* (P. Karst.) Donk, while the taxon *X.guttulata* has a close relationship with *X.pruinosum* with strong support (97/100/0.96).

The BLAST result of four new species for the closest top 10 taxa and their corresponding parameters are given (Table 2).

**Table 2.** The BLAST result of four new species for the closest top 10 taxa and their corresponding parameters.

Scientific Name	Specimens	Sequence Number	Max score	Total score	Query cover	E value	Per. Ident.	Acc. len.	Accession
*Clavulinopsiswumengshanensis*	CLZhao 29612	PQ408629	1123	1123	100%	0	100%	638	PQ408629
			734	734	71%	0	97.24%	431	PQ515870
			448	448	44%	6e-121	96.68%	295	PQ453614
			911	911	93%	0	95.94%	573	ON943319
			972	972	100%	0	95.60%	627	PQ346233
			970	970	100%	0	95.59%	626	PQ346239
			976	976	100%	0	95.57%	624	PQ346237
			966	966	100%	0	95.43%	653	PQ453611
			966	966	100%	0	95.43%	632	PQ346229
			966	966	100%	0	95.43%	627	PQ346265
*Clavulinopsiswumengshanensis*	CLZhao 29612	PQ408634	1024	1024	100%	0	100.00%	638	PQ408629
			896	896	100%	0	95.85%	643	PQ408630
			885	885	99%	0	95.99%	627	PQ346233
			881	881	100%	0	95.32%	624	PQ346237
			880	880	99%	0	95.81%	611	PQ453612
			880	880	99%	0	95.81%	653	PQ453611
			880	880	99%	0	95.81%	626	PQ346239
			880	880	99%	0	95.81%	632	PQ346229
			880	880	99%	0	95.81%	627	PQ346265
			878	878	99%	0	95.63%	590	PQ453613
*Henningsomycesbambusae*	CLZhao 33088	PQ408628	1282	1282	100%	0	100.00%	694	PQ408628
			1253	1253	98%	0	99.85%	694	PQ408626
			1223	1223	97%	0	99.26%	697	PQ408627
			1081	1081	96%	0	95.71%	701	LC774058
			1077	1077	96%	0	95.56%	733	PP849903
			1072	1072	96%	0	95.41%	732	MG707601
			1059	1059	96%	0	951%	745	MK607599
			985	985	89%	0	95.22%	643	AB847016
			950	950	89%	0	94.38%	622	AY571057
			813	813	90%	0	903%	652	OQ872100
*Xenasmabisterigmatae*	CLZhao 32542	PQ408631	1138	1138	100%	0	100.00%	616	PQ408631
			1040	1040	99%	0	97.55%	611	PQ408632
			846	846	92%	0	93.58%	634	MT537020
			758	676	83%	0	92.83%	676	MT571671
			597	597	60%	6e-166	95.48%	411	LR819650
			597	597	60%	6e-166	95.48%	411	LR602855
			508	508	72%	3e-139	87.25%	636	JF691144
			503	503	91%	1e-137	86.96%	675	PQ408633
			492	492	59%	3e-134	90.98%	638	OM009268
			431	431	60%	6e-116	87.47%	417	LR819360
*Xenasmaguttulata*	CLZhao 32193	PQ408633	1247	1247	100%	0	100.00%	675	PQ408633
			737	737	91%	0	88.36%	636	JF691144
			634	634	91%	5e-177	85.53%	638	OM009268
			592	592	55%	3e-164	94.97%	417	LR819360
			592	592	55%	3e-164	94.97%	417	LR602599
			586	586	55%	1e-162	94.71%	417	LR602541
			586	586	55%	1e-162	94.71%	417	LR819292
			558	558	55%	3e-154	93.39%	417	LR602585
			558	558	55%	3e-154	93.39%	417	LR819344
			507	507	47%	1e-138	95.34%	417	MT852420

### ﻿Taxonomy

#### ﻿ Clavulinopsis wumengshanensis

Taxon classificationFungiAgaricalesClavariaceae

S.Y. He., H.M. Zhou & C.L. Zhao sp. nov.

928D9055-3FB7-5CF0-87EF-61BF521B8F8E

855906

Figs 4 , 5

##### Diagnosis.

*Clavulinopsiswumengshanensis* differs from *C.aurantiocinnabarina* by buff-yellow to straw-yellow basidiomata and thick-walled, subglobose, and longer basidiospores (7–8.5 µm vs. 5.6–7.1 µm).

##### Holotype.

China • Yunnan Province, Zhaotong, Yiliang County, Wumengshan National Nature Reserve, GPS coordinates 27°30'N, 104°12'E, evel. 1710 m asl., on the ground, leg. C.L. Zhao, 12 July 2023, CLZhao 29612 (SWFC).

##### Etymology.

*Wumengshanensis* (Lat.) refers to the locality “Wumengshan National Nature Reserve” of the holotype.

##### Basidiomata.

Basidiomes annual, clavarioid, without odor or taste when fresh, up to 8 cm long, 4 mm wide, and 400–800 µm thick. Fertile part subcylindrical to fusiform, occasionally slightly curved or flexuous and with a distinct longitudinal depression, buff-yellow (4A4) to straw-yellow (3A/B3) when dry. Apex rounded or obtuse acute when mature, concolourous or slightly paler.

**Figure 4.**
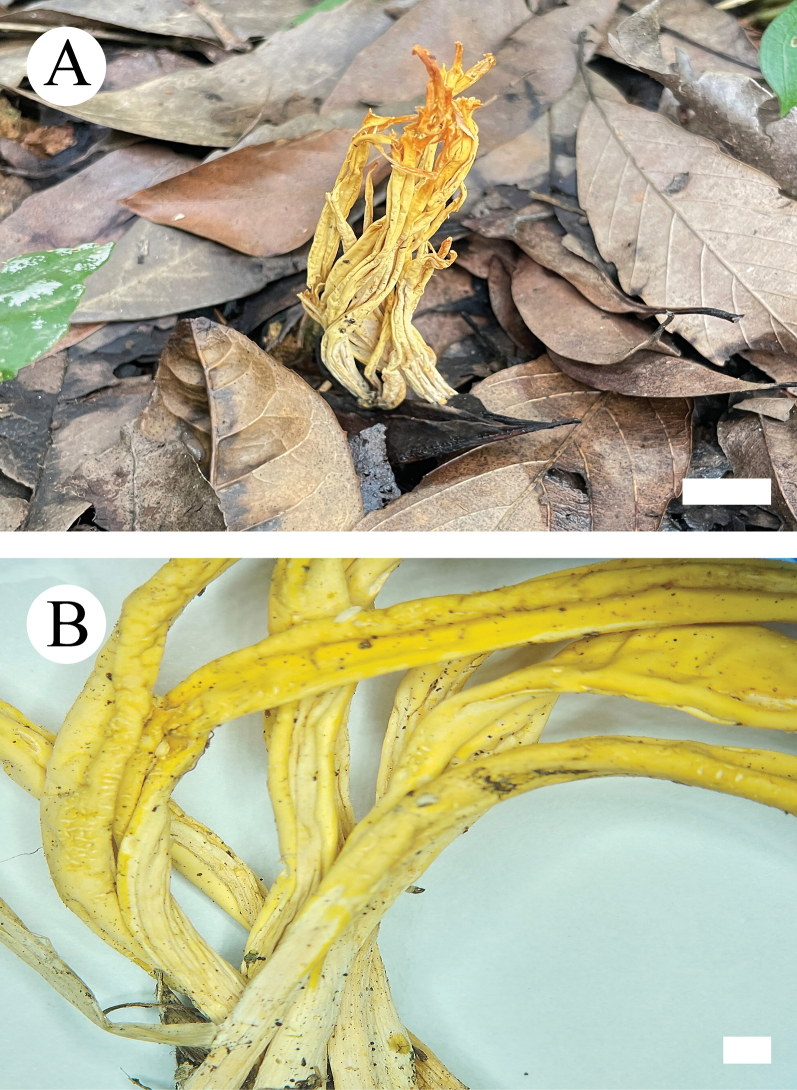
Basidiomata of *Clavulinopsiswumengshanensis* (holotype, CLZhao 29612). Scale bars: 1 cm (**A**); 1 mm (**B**).

##### Hyphal structure.

Monomitic, generative hyphae with clamp connections, hyaline, thick-walled, parallel, interwoven, 2–3 µm in diam, some inflated to 13 µm in diam, IKI–, CB–; tissues unchanged in KOH. Cystidia absent. Basidia clavate to subcylindrical, hyaline, thin-walled, with four sterigmata and a basal clamp connection, 35–48.5 × 9–11.5 µm; basidioles in shape similar to basidia, with several guttules.

**Figure 5.**
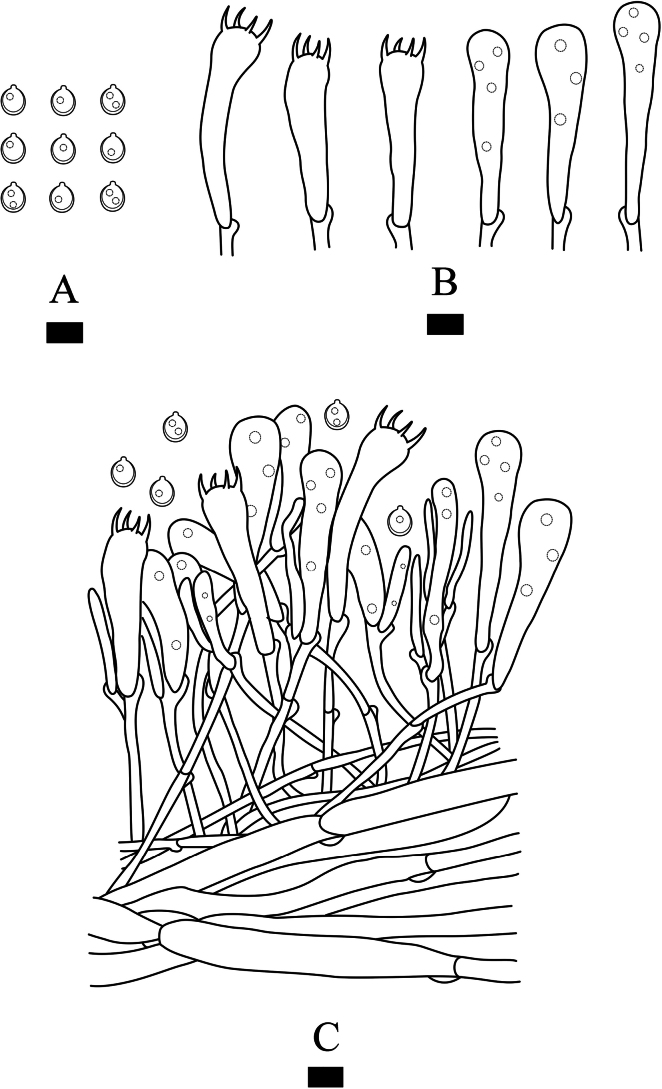
Microscopic structures of *Clavulinopsiswumengshanensis* (holotype, CLZhao 29612) **A** basidiospores **B** basidia and basidioles **C** a section of basidiomata. Scale bars: 10 µm (**A–C**).

##### Spores.

Basidiospores subglobose with a distinct apiculus, hyaline, thick-walled, smooth, with several guttules, IKI–, CB–, 7–8.5(–5) × (5.5–)6–7.5 µm, L = 7.59 µm, W = 6.54 µm, Q = 1.14–1.16 (n = 60/2).

##### Additional specimen examined

**(*paratype*).** China • Yunnan Province, Zhaotong, Yiliang County, Wumengshan National Nature Reserve, GPS coordinates 27°30'N, 104°12'E, evel. 1710 m asl., on the ground, leg. C.L. Zhao, 12 July 2023, CLZhao 29651 (SWFC).

##### Notes.

Based on the ITS + LSU analysis (Fig. 1), the result showed that the new species *Clavulinopsiswumengshanensis* is grouped with *C.aurantiocinnabarina* (Schwein.) Corner. However, *C.aurantiocinnabarina* differs from *C.wumengshanensis* by its thin-walled basidiospores and narrower basidia (5.2–7.1 µm vs. 9–11.5 µm, Petersen 1978).

#### ﻿ Henningsomyces bambusae

Taxon classificationFungiAgaricalesClavariaceae

S.Y. He, H.M. Zhou & C.L. Zhao sp. nov.

3AEA4B0B-29FF-5A1F-8345-48B96967D3DB

855907

Figs 6 , 7

##### Diagnosis.

*Henningsomycesbambusae* differs from *H.candidus* by its globose to subglobose and wider basidiospores (6.5–8.5 µm vs. 4–5 µm).

##### Holotype.

China • Yunnan Province, Zhaotong, Wumengshan National Nature Reserve, GPS coordinates 27°29'N, 103°55'E, evel. 1900 m asl., on dead bamboo, leg. C.L. Zhao, 18 September 2023, CLZhao 33088 (SWFC).

##### Etymology.

*Bambusae* (Lat.) refers to the host genus *Bambusa*.

##### Basidiomata.

Basidiomes forming loosely scattered colonies, short cylindric to tubular, sessile, up to 1–1.6 cm in length, 0.5–1 cm diam; external surface white (60) to cream (4A2/3), finely tomentose when dry; inner surface covered by a deeply concave, smooth hymenium.

**Figure 6.**
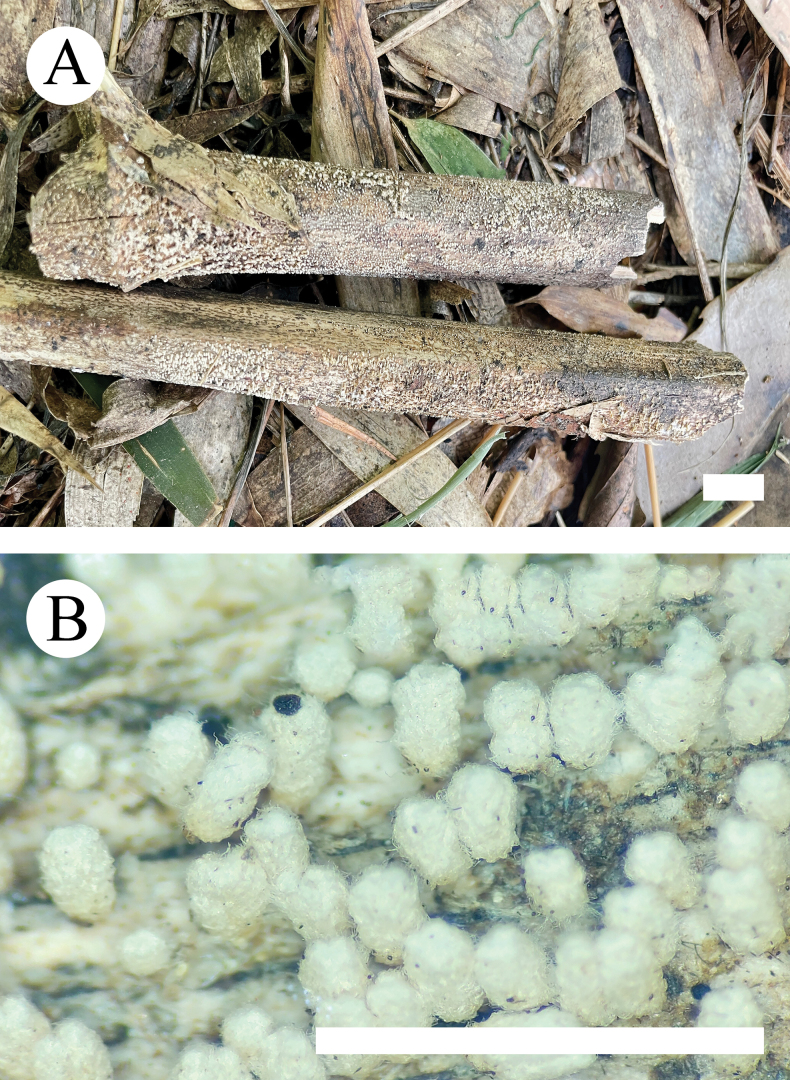
Basidiomata of *Henningsomycesbambusae* (holotype, CLZhao 33088). Scale bars: 1 cm (**A**); 1 mm (**B**).

##### Hyphal structure.

Monomitic, generative hyphae with clamp connections and simple septa, thin- to thick-walled, IKI–, CB–; tissues unchanged in KOH. External hyphae slightly contorted, widest at the base, slightly tapering towards the obtusely rounded apex, hyaline, thick-walled to solid, occasionally with simple septa, frequently unbranched, dextrinoid, CB–, 3–4 μm diam; tramal hyphae hyaline, thin-walled, with clamp connections, IKI–, 2.5–3 µm diam. Cystidia absent. Basidia cylindrical to subcylindrical, hyaline, thin-walled, with two sterigmata and a basal clamp connection, 19.5–22.5 × 7–8.5 µm; basidioles in shape similar to basidia, but slightly smaller.

**Figure 7.**
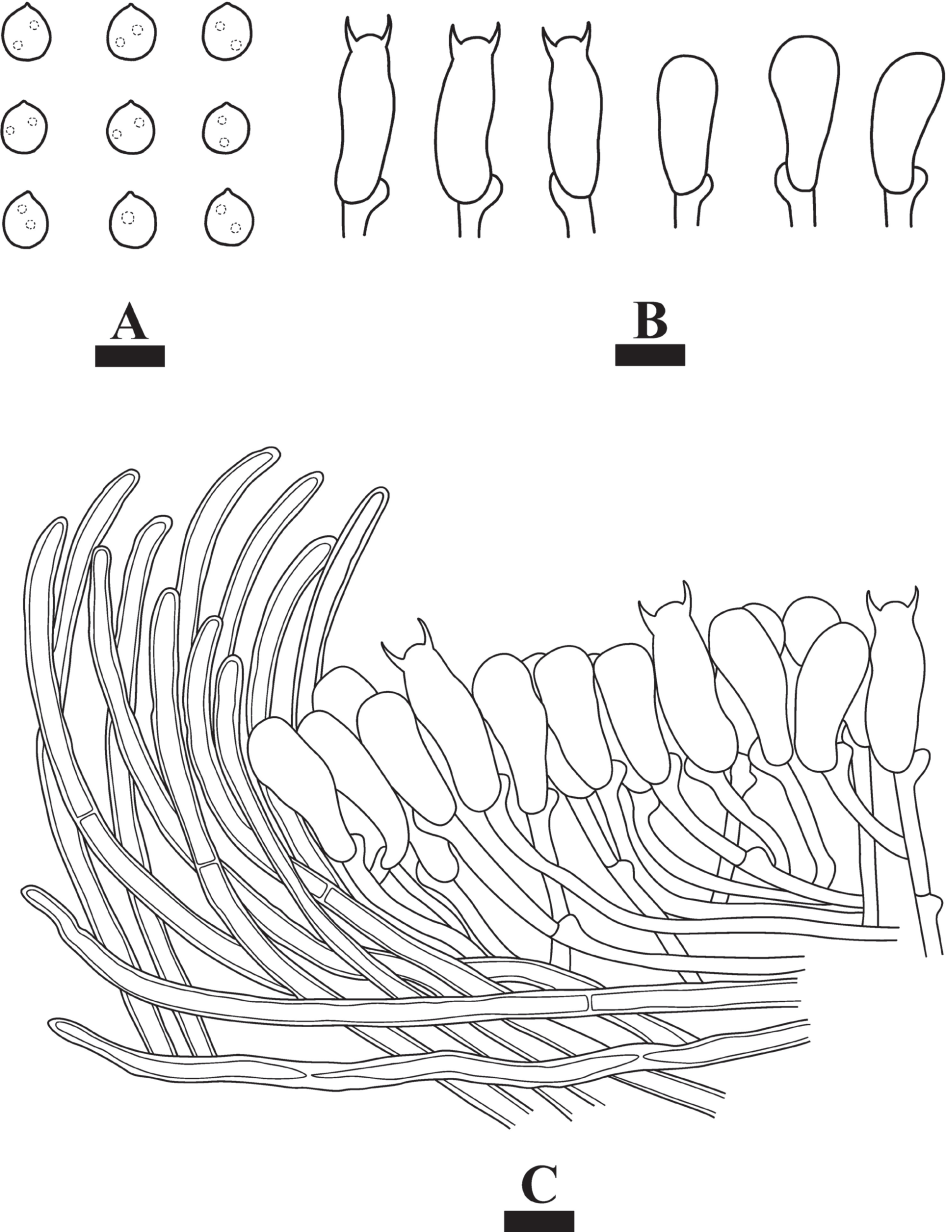
Microscopic structures of *Henningsomycesbambusae* (holotype, CLZhao 33088) **A** basidiospores **B** basidia and basidioles **C** a section of basidiomata. Scale bars: 10 µm (**A–C**).

##### Spores.

Basidiospores globose to subglobose, hyaline, thin-walled, smooth, with guttules, IKI–, CB–, 6.5–8.5(–9) × (6–)6.5–8.5(–9) µm, Lm = 7.61 µm, Wm = 7.45 µm, Q = 1.02–1.04 (n = 60/2).

##### Additional specimens examined

**(*paratypes*).** China • Yunnan Province, Zhaotong, Wumengshan National Nature Reserve, GPS coordinates 27°29'N, 103°55'E, evel. 1900 m asl., on a dead bamboo, leg. C.L. Zhao, 18 September 2023, CLZhao 33024; CLZhao 33085 (SWFC).

##### Notes.

Based on the ITS analysis (Fig. 2), the new taxon *Henningsomycesbambusae* grouped within the genus *Henningsomyces* and formed a sister group with *H.candidus* (Pers.) Kuntze. However, *H.candidus* can be delimited from *H.bambusae* by its narrower basidiospores (4–5 μm vs. 6–8 μm; Gilbertson and Blackwell 1985).

#### ﻿ Xenasma bisterigmatae

Taxon classificationFungiRussulalesXenasmataceae

S.Y. He, H.M. Zhou & C.L. Zhao sp. nov.

3AAD8925-01A9-5D53-8809-965A9559D50E

855908

Figs 8 , 9

##### Diagnosis.

*Xenasmabisterigmatae* differs from *X.rimicola* by its ellipsoid to subglobose and larger basidiospores (8.5–10.5 × 4.5–7 µm vs. 10–12.5 × 8–10.5 µm), two-sterigmata basidia.

##### Holotype.

China • Yunnan Province, Zhaotong, Wumengshan National Nature Reserve, GPS coordinates 27°29'N, 103°55'E, evel. 1900 m asl., on fallen angiosperm branch, leg. C.L. Zhao, 29 August 2023, CLZhao 32542 (SWFC).

##### Etymology.

*Bisterigmatae* (Lat.) refers to two-sterigmata basidia of the holotype.

##### Basidiomata.

Basidiomes annual, resupinate, smooth, without odor or taste when fresh, up to 6.5 cm long, 2.3 cm wide, and 100 µm thick. Hymenial surface smooth, membranaceous, ash-grey (19C2) when dry; sterile margin indistinct.

**Figure 8.**
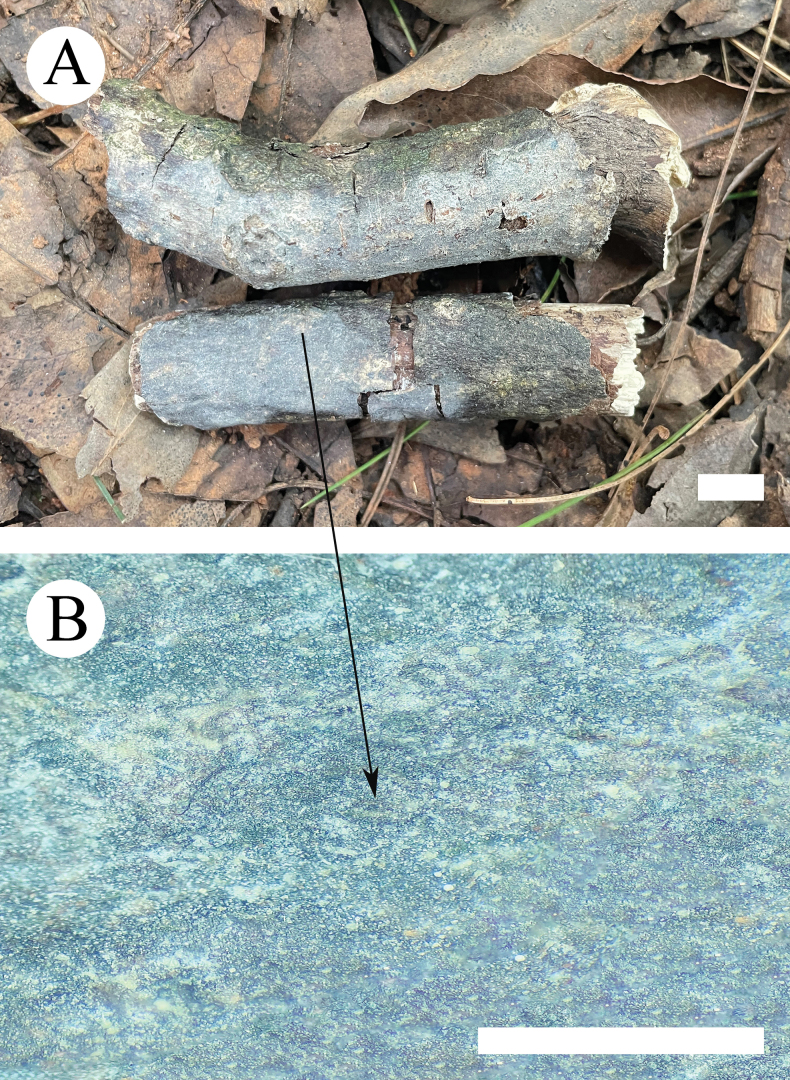
Basidiomata of *Xenasmabisterigmatae* (holotype, CLZhao 32542). Scale bars: 1 cm (**A**); 1 mm (**B**).

##### Hyphal structure.

Monomitic, generative hyphae with clamp connections, thin-walled, branched, interwoven, with dense crystal, 3.2–3.5 µm diam, IKI–, CB–; tissues unchanged in KOH. Cystidia abundant, tubular with obtuse apex, with slightly thick walls in the basal part that appears frequently collapsed, often with an apical amorphous globule, 64.5–93 × 5.5–7.5 µm. Cystidioles absent. Basidia clavate to subcylindrical, thin-walled, with two sterigmata and a basal clamp connection, with several guttules, 21.5–28 × 10–12 µm; basidioles in shape similar to basidia, but slightly smaller.

**Figure 9.**
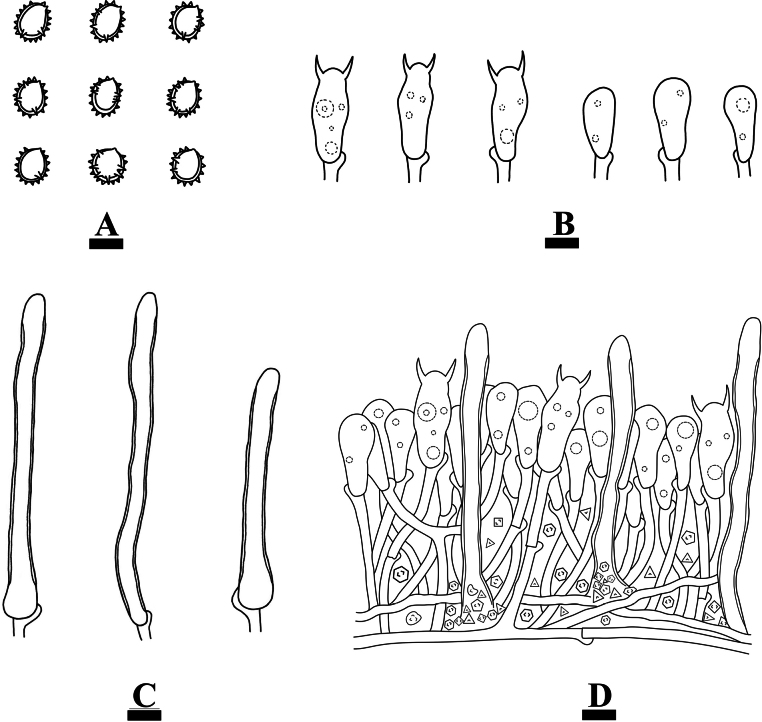
Microscopic structures of *Xenasmabisterigmatae* (holotype, CLZhao 32542) **A** basidiospores **B** basidia and basidioles **C** cystidia **D** a section of basidiomata. Scale bars: 10 µm (**A–C**).

##### Spores.

Basidiospores ellipsoid to subglobose, thick-walled, verrucose, IKI–, CB–, 10–12.5 × 8–10.5(–11) µm, Lm = 11.15 µm, Wm = 9.3 µm, Q = 1.19–1.27 (n = 60/2).

##### Additional specimens examined

**(*paratype*).** China • Yunnan Province, Zhaotong, Wumengshan National Nature Reserve, GPS coordinates 27°29'N, 103°55'E, evel. 1900 m asl., on fallen angiosperm branch, leg. C.L. Zhao, 29 August 2023, CLZhao 32600 (SWFC).

##### Notes.

Based on the ITS analysis (Fig. 3), the new species *Xenasmabisterigmatae* was clustered with *X.rimicola* (P. Karst.) Donk. However, *X.rimicola* differs from *X.bisterigmatae* by its four-sterigmata basidia (Cunningham 1963).

#### ﻿ Xenasma guttulata

Taxon classificationFungiRussulalesXenasmataceae

S.Y. He, H.M. Zhou & C.L. Zhao sp. nov.

B1138407-B8F0-575D-B16D-1BCA1F567466

855909

Figs 10 , 11

##### Diagnosis.

*Xenasmaguttulata* differs from *X.pruinosum* by its membranaceous basidiomata with a white to cream hymenial surface and larger basidiospores (6–7 × 3–4 µm vs. 7–9 × 5.5–7.5 µm).

##### Holotype.

China • Yunnan Province, Zhaotong, Wumengshan National Nature Reserve, GPS coordinates 27°29'N, 103°55'E, evel. 1900 m asl., on a fallen angiosperm branch, leg. C.L. Zhao, 29 August 2023, CLZhao 32193 (SWFC).

##### Etymology.

*Guttulata* (Lat.) refers to the basidiospores with guttules in the holotype.

##### Basidiomata.

Basidiomes annual, resupinate, closely adnate, without odor or taste when fresh, up to 2.5 cm long, 1.5 cm wide, and 100 µm thick. Hymenial surface smooth, membranaceous, white (60) to cream (4A2/3) when dry; sterile margin indistinct.

**Figure 10.**
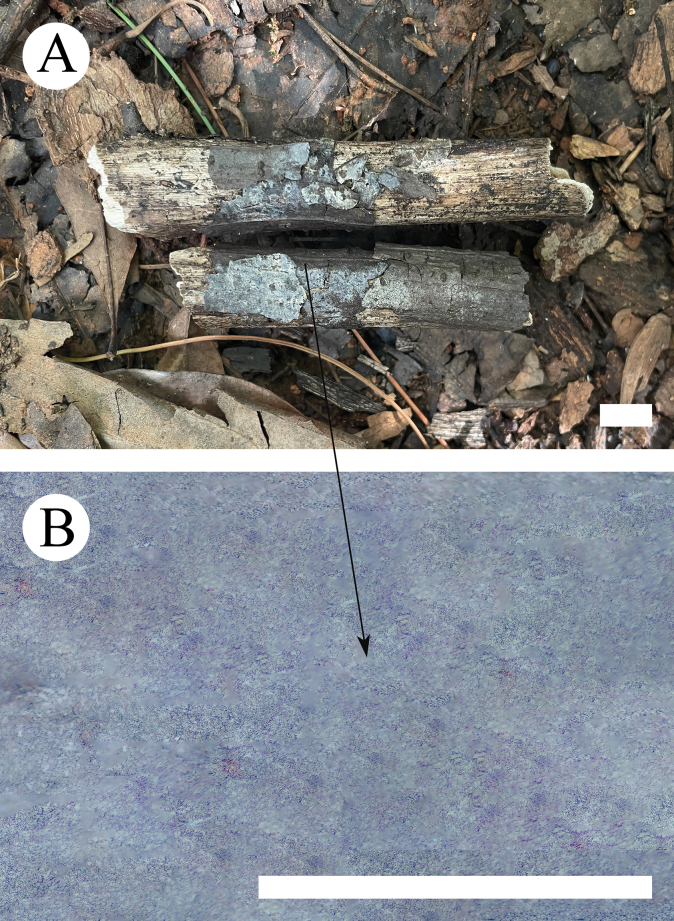
Basidiomata of *Xenasmaguttulata* (holotype, CLZhao 32193). Scale bars: 1 cm (**A**); 1 mm (**B**).

##### Hyphal structure.

Monomitic, generative hyphae with clamp connections, thin-walled, occasionally branched, interwoven, with dense crystal, 3.3–3.5 µm diam, IKI–, CB–; tissues unchanged in KOH. Cystidia abundant, cystidia tubular with obtuse apex, 75–91.5 × 4–8.5 µm, with slightly thick walls in the basal part that appears frequently collapsed, often with an apical amorphous globule. Cystidioles absent. Basidia clavate to subcylindrical, thin-walled, with two sterigmata and a basal clamp connection, 18.5–24 × 7.5–11 µm; basidioles in shape similar to basidia, but slightly smaller.

**Figure 11.**
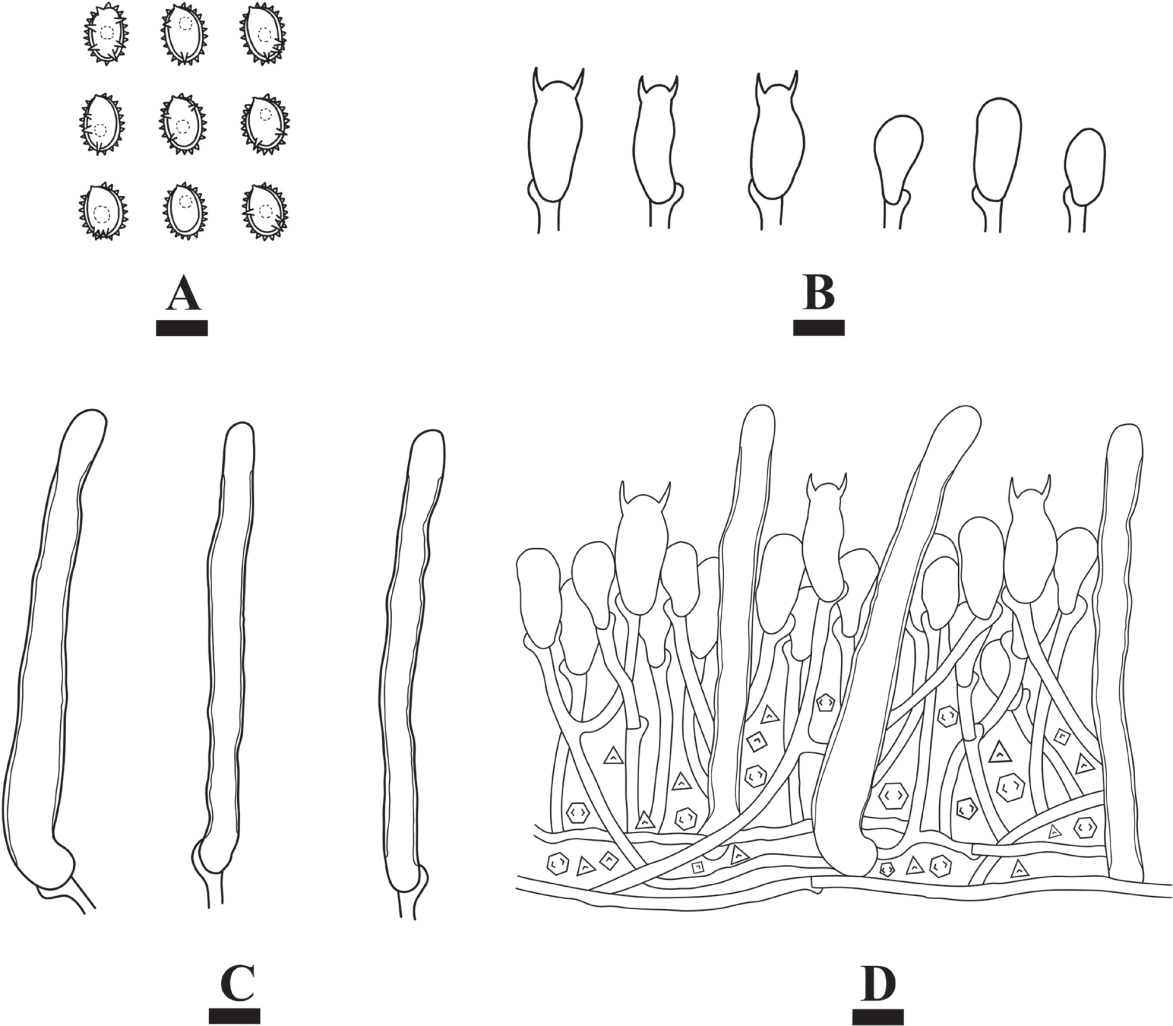
Microscopic structures of *Xenasmaguttulata* (holotype, CLZhao 32193) **A** basidiospores **B** basidia and basidioles **C** cystidia **D** a section of basidiomata. Scale bars: 10 µm (**A–C**).

##### Spores.

Basidiospores ellipsoid to narrowly ellipsoid, thick-walled, verrucose, with several guttules, IKI–, CB–, 7–9 × 5.5–7.5 µm, Lm = 8.05 µm, Wm = 6.52 µm, Q = 1.23 (n = 30/1).

##### Notes.

Based on the ITS analysis (Fig. 3), the new species *Xenasmaguttulata* was clustered with *X.pruinosum* (Pat.) Donk. However, *X.pruinosum* can be distinguished from *X.guttulata* by its smaller basidiospores (6–7 × 3–4 µm vs. 7–9 × 5.5–7.5; Donk 1957; Bernicchia and Gorjón 2010).

## ﻿Discussion

The multilocus (ITS+LSU) analysis (Fig. 1) showed that the new species *Clavulinopsiswumengshanensis* groups with *C.aurantiocinnabarina* (Schwein.) Corner. However, *C.aurantiocinnabarina* differs from *C.wumengshanensis* by its thin-walled basidiospores and narrower basidia (5.2–7.1 µm vs. 9–11.5 µm) (Petersen 1978). Morphologically, *Clavulinopsisfusiformis* (Sowerby) Corner and *C.incarnata* P. Zhang & Jun Yan are similar to *C.wumengshanensis* by both having smooth, globose to subglobose basidiospores (Keleş and Kaya 2021; Yan et al. 2023). However, *C.fusiformis* differs in its pale yellow basidiomata, and *C.incarnata* differs in its pinkish basidiomata (Keleş and Kaya 2021; Yan et al. 2023).

Based on the ITS locus phylogenetic analysis (Fig. 2), the new taxon *Henningsomycesbambusae* forms a sister group with *H.candidus* (Pers.) Kuntze. However, *H.candidus* can be delimited from *H.bambusae* by its narrower basidiospores (4–5 μm vs. 6–8 μm, Gilbertson and Blackwell 1985). Morphologically, *Henningsomyceshengduanensis* S.L. Liu & L.W. Zhou and *H.minimus* (Cooke and W. Phillips) Kuntze are similar to *H.bambusae* by both having thin-walled, smooth, subglobose basidiospores (Wei and Qin 2009; Liu et al. 2023). However, *H.hengduanensis* differs in its cream basidiomata and broadly clavate basidia with four sterigmata (Liu et al. 2023); *Henningsomycesminimus* differs in its shorter (11–15 μm vs. 19.5–22.5 μm), broadly clavate basidia with four sterigmata (Wei and Qin 2009).

Based on the combined ITS locus phylogeny (Fig. 3), *X.rimicola* (P. Karst.) Donk differs from the new species *X.bisterigmatae* by its four-sterigmata basida (Cunningham 1963); *X.pruinosum* (Pat.) Donk can be distinguished from the new species *X.guttulata* by its smaller basidiospores (6–7 × 3–4 µm vs. 7–9 × 5.5–7.5, Donk 1957; Bernicchia and Gorjón 2010). Morphologically, *X.pruinosum* is similar to *X.bisterigmatae* by having tubular cystidia with an apical amorphous globule and slightly thick walls in the basal part (Donk 1957; Bernicchia and Gorjón 2010). However, *X.pruinosum* differs in its smaller basidiospores (6–7 × 3–4 µm vs. 7–9 × 5.5–7.5 µm; Donk 1957; Bernicchia and Gorjón 2010). *Xenasmarimicola* is similar to *X.guttulata* by having tubular cystidia and slightly thick walls in the basal part (Donk 1957). However, *X.rimicola* differs in its subclavate basidia with four sterigmata (Donk 1957).

Yunnan Province is located in the southwest of China. The climate in Yunnan offers conducive environments for the speciation and diversification of various life forms, and this climatic diversity in Yunnan creates varied landscapes with multiple habitats, resulting in a high species diversity, with over 6,000 recorded fungal species up to now (Wang and Cai 2022; Dong et al. 2024; Wang et al. 2024; Zhou et al. 2024b). Therefore, focusing on the diversity of fungi in the Yunnan-Guizhou Plateau of China is of great significance. Based on the present study, the results not only enrich the species diversity of fungi worldwide but also contribute to the branches of the fungal tree of life.

## Supplementary Material

XML Treatment for Clavulinopsis wumengshanensis

XML Treatment for Henningsomyces bambusae

XML Treatment for Xenasma bisterigmatae

XML Treatment for Xenasma guttulata

## ﻿Additional information

### Author ORCIDs

Siyuan He https://orcid.org/0009-0005-2478-0624

Lu Wang https://orcid.org/0009-0004-6274-5953

Hongmin Zhou https://orcid.org/0000-0002-0724-5815

### Data availability

All of the data that support the findings of this study are available in the main text or Supplementary Information.
